# Folate deficiency as predisposing factor for childhood leukaemia: a review of the literature

**DOI:** 10.1186/s12263-017-0560-8

**Published:** 2017-06-02

**Authors:** Catia Daniela Cantarella, Denise Ragusa, Marco Giammanco, Sabrina Tosi

**Affiliations:** 1 Department of Experimental Biomedicine and Clinical Neurosciences, University of Palermo, Palermo, Italy; 2 Division of Biosciences, College of Health and Life Sciences, Institute of Environment, Health and Societies, Brunel University London, Uxbridge, UK

**Keywords:** Folic acid, Folates, Genomic health, Childhood leukaemia, Cancer, DNA methylation

## Abstract

**Background:**

Folic acid and its derivates, known as folates, are chemoprotective micronutrients of great interest because of their essential role in the maintenance of health and genomic integrity. The supplementation of folic acid during pregnancy has long been known to reduce the risk of neural tube defects (NTDs) in the foetus. Folate metabolism can be altered by many factors, including adequate intake through diet. Folate deficiency can compromise the synthesis, repair and methylation of DNA, with deleterious consequences on genomic stability and gene expression. These processes are known to be altered in chronic diseases, including cancer and cardiovascular diseases.

**Main body:**

This review focuses on the association between folate intake and the risk of childhood leukaemia. Having compiled and analysed studies from the literature, we show the documented effects of folates on the genome and their role in cancer prevention and progression with particular emphasis on DNA methylation modifications. These changes are of crucial importance during pregnancy, as maternal diet has a profound impact on the metabolic and physiological functions of the foetus and the susceptibility to disease in later life. Folate deficiency is capable of modifying the methylation status of certain genes at birth in both animals and humans, with potential pathogenic and tumorigenic effects on the progeny. Pre-existing genetic polymorphisms can modify the metabolic network of folates and influence the risk of cancer, including childhood leukaemias. The protective effects of folic acid might be dose dependent, as excessive folic acid could have the adverse effect of nourishing certain types of tumours.

**Conclusion:**

Overall, maternal folic acid supplementation before and during pregnancy seems to confer protection against the risk of childhood leukaemia in the offspring. The optimal folic acid requirements and supplementation doses need to be established, especially in conjunction with other vitamins in order to determine the most successful combinations of nutrients to maintain genomic health and wellbeing. Further research is therefore needed to uncover the role of maternal diet as a whole, as it represents a main factor capable of inducing permanent changes in the foetus.

## Background

Nutrition represents one of the leading preventable risk factors in the development of cancer, accounting for nearly 10% of total cases in the UK [1]. The concept of chemoprevention in the insurgence of cancer was first introduced by Sporn [2, 3] and has since been employed in the attempt to arrest, retard or reverse tumorigenic processes by the use of biological and nutritional compounds such as phytochemicals (e.g. carotenoids, allyl sulphur compounds, glucosinolates, isothiocyanates and polyphenols) and vitamins. Diet and the assimilation of micronutrients, therefore, have a substantial impact on health and disease.

Folic acid (or vitamin B9) and its derivatives, collectively known as folates, are chemoprotective micronutrients of great interest belonging to the B vitamin group. They are water-soluble vitamins that function as co-factors in a variety of enzymatic reactions within the cell. Folates are naturally found in leafy vegetables, eggs, legumes, bran and dry fruit, whereas the synthetic form, which has a higher bioavailability, is added as a food fortifier in cereal grain products or used as a dietary supplementation [4, 5].

**Fig. 1:**
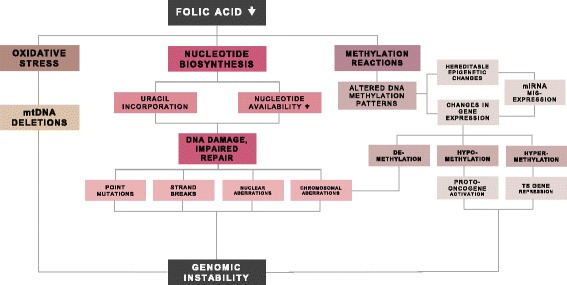
The relationship between folic acid deficiency and the genomic instability. Low levels of folic acid interfere with the normal biological functions of the cell, compromising the genomic stability for both nuclear and mitochondrial DNA. Folic acid deficiency affects the synthesis of nucleotides, causing DNA damage that cannot be repaired efficiently because of an overall decrease in the nucleotide availability. As methyl donors, the unavailability of folates can alter DNA methylation, causing changes in gene expression and compromising the integrity of chromosomes. Deprivation of folic acid also causes oxidative stress in the cell with consequences affecting the integrity of mitochondrial DNA

Folate is essential for the correct functioning of the human body and the maintenance of genomic integrity. Within the cell, it participates in two types of reactions, biosynthesis of nucleotides and methylation reactions, which are required in the fundamental biological processes of DNA synthesis, DNA repair and DNA methylation [6–8]. Folic acid is also needed for correct functioning of mitochondria and maintenance of mitochondrial DNA (mtDNA) [9] (Fig. 1).

Folate deficiency is associated with several disorders such as neural tube defects (NTDs) and malformations in the developing foetus, as well as cardiovascular diseases, depression and Alzheimer’s disease in adults [10–12]. Given its role in the maintenance of genomic stability, insufficient folic acid also appears to be involved in the insurgence of cancer [13]. In addition to the above, there is evidence that folic acid deficiency during pregnancy could represent a risk factor for the development of childhood leukaemia in the offspring.

The chemoprotective properties of folic acid with respect to cancer initiation and progression will be explained. In particular, the effects of folate deficiency on genomic health and its potential impact on the insurgence of childhood leukaemia will be discussed. Several studies and findings have been compiled to provide a comprehensive view of current research covering these aspects:

1. The association between folate intake and the risk of childhood leukaemia, by considering studies on the efficacy of folic acid supplementation before or during pregnancy in preventing the disease in the offspring
2. The role of folic acid in influencing the methylation patterns of DNA, an inheritable epigenetic regulatory mechanism capable of altering gene expression in the progeny

## The biological role of folates in the cell

**Fig. 2:**
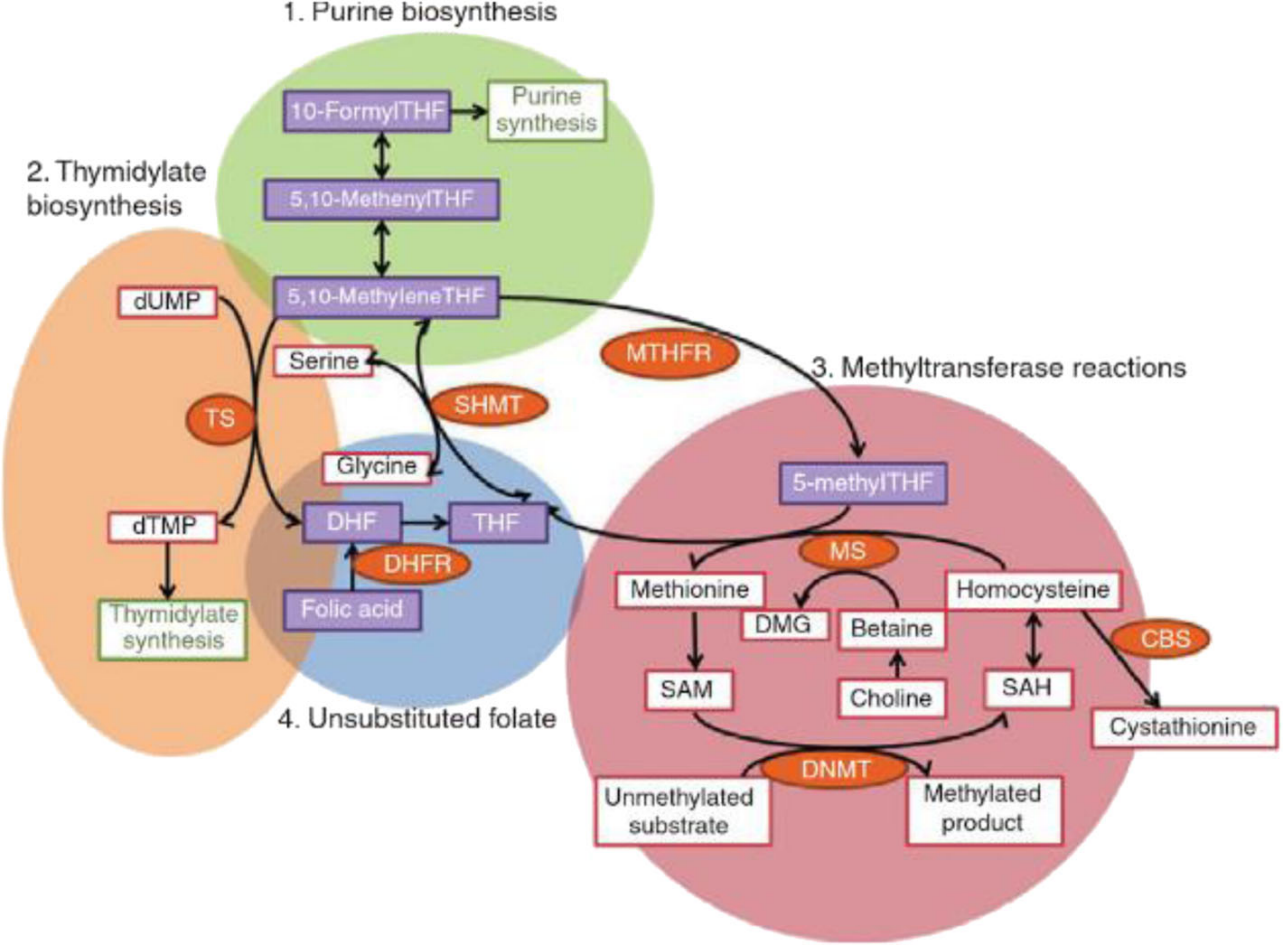
Intracellular network of reactions in the metabolism of folates. A variety of related compounds derived from folic acid have specific biochemical functions. Folic acid is first converted into dihydrofolate (*DHF*) and subsequently into tetrahydrofolate (*THF*) by the enzyme DHF reductase (*DHFR*). 5,10-methyleneTHF and 10-formylTHF are responsible for the synthesis of purines; 5,10-methyleneTHF also mediates the conversion of dUMP to dTMP for the synthesis of thymidine, catalysed by the enzyme thymidine synthase (*TS*). 5-methylTHF is involved for methylation reactions, particularly in the conversion of homocysteine to methionine for the formation of SAM, a major methyl donor for DNA (figure taken from [6])

Folates are involved in the cellular one-carbon metabolism, acting as one-carbon carriers for the transfer of methyl groups. The metabolically active form of folic acid is tetrahydrofolate (THF), which can be converted into other structurally related molecules, each having a specific function and forming a complex network of enzymatic reactions (Fig. 2).

### Nucleotide biosynthesis

Folates are responsible for the synthesis of purines and pyrimidines for the correct assembly of DNA and RNA. In particular, they mediate the only known reaction for the de novo synthesis of thymidine, which consists in the conversion of deoxyuridine monophosphate (dUMP) to deoxythymidine monophosphate (dTMP) by the transfer of a methyl group [14, 15].

### Methylation reactions

As methyl group donors, folates are involved in methylation reactions including DNA methylation, a major epigenetic process capable of influencing gene expression. Specific loci known as CpG islands are methylated through the modification of cytosine to form 5-methylcytosine by an enzymatic reaction involving the transfer of a methyl group from S-adenosylmethionine (SAM) [16]. Folates are involved in the conversion of homocysteine to methionine required for the formation of SAM. This is achieved by a one-carbon transfer to vitamin B12, which will then be used for the methylation of homocysteine to methionine. By the addition of adenine to methionine, SAM is formed, which is the main methyl donor for DNA [17].

## Folate deficiency and genomic damage

**Fig. 3:**
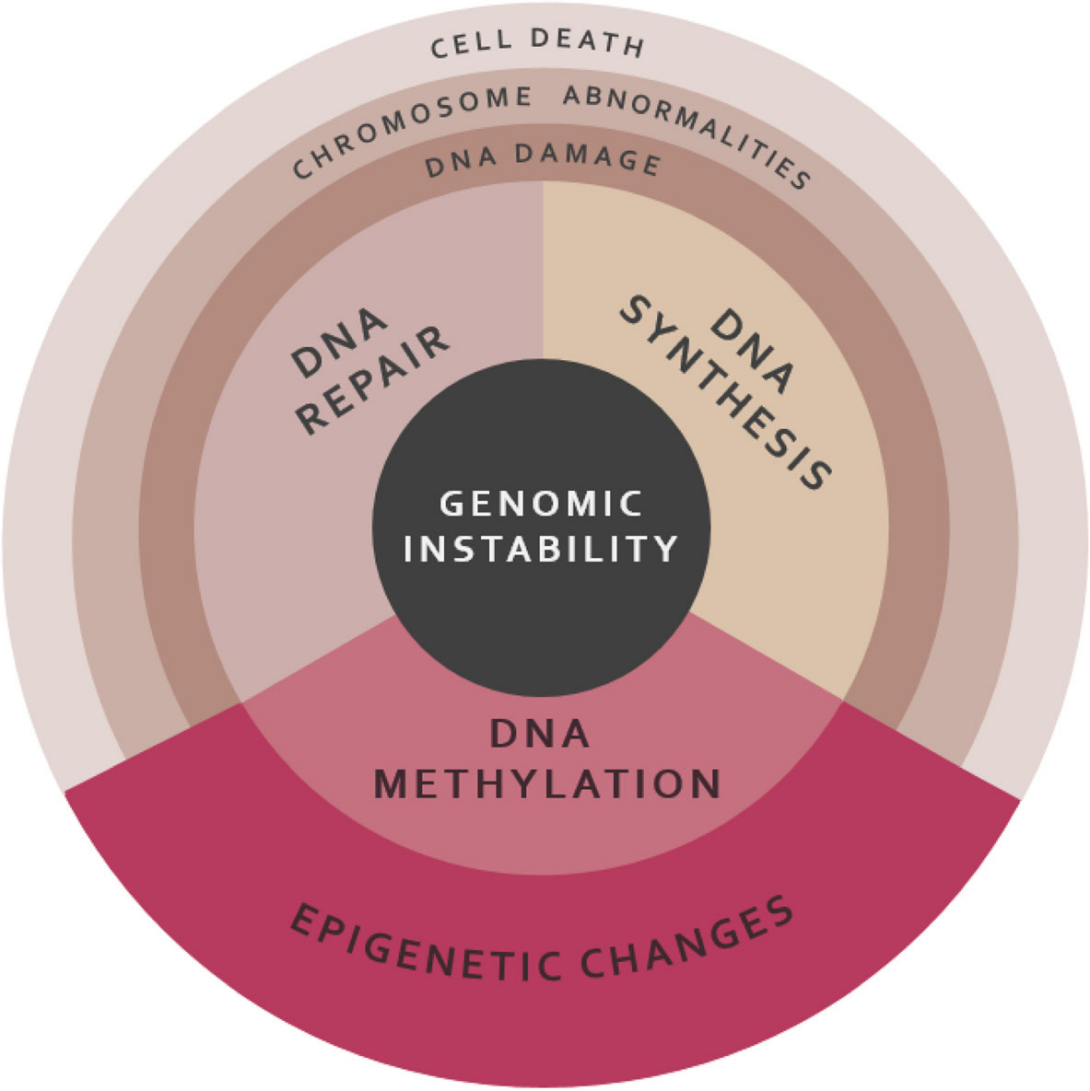
Processes and consequences contributing to the genomic instability of the cell. Altered DNA synthesis, DNA repair and DNA methylation compromise the genomic stability of the cell. DNA damage and chromosomal abnormalities result from an incorrect assembly of DNA and the inability to repair errors efficiently, potentially affecting the next generation of cells or leading to cell death. If DNA methylation is disrupted, epigenetic changes affecting gene expression can occur, including incorrect methylation patterns or changes in chromatin structure

Abnormalities in nucleotide biosynthesis and methylation reactions are capable of affecting DNA synthesis, DNA repair and DNA methylation, potentially leading to genomic instability of the cell (Fig. 3).

In conditions of folic acid depletion, the conversion of dUMP cannot proceed, leading to its abnormal intracellular accumulation and misincorporation in the DNA instead of thymine [18]. Excessive uracil content causes point mutations, single- and double-strand DNA breaks, chromosome breaks and formation of micronuclei [19, 20]. The inability to provide nucleotides adequately renders DNA synthesis inefficient, compromising the regenerative power of tissues [21] and the ability to repair DNA efficiently [22].

Disruption of methylation reactions is not limited to altered gene expression. Demethylation of centromeres causes structural and functional aberrations within the chromosome, notably during mitosis, leading to abnormal chromosome segregation and aneuploidy [23]. Low folate has also been shown to alter the expression of microRNAs (miRNAs) due to abnormal DNA methylation [24]. miRNAs are non-coding oligonucleotide RNAs detaining an important role in gene regulation. When abnormally expressed, they can gain oncogenic properties and initiate tumorigenesis [25] and NTDs [26]. Studies on the expression profile of miRNAs have been conducted to uncover a possible role in leukaemogenesis [27] because of their regulatory function in haematopoiesis [28]. The data collected from in vitro experiments on DNA damage and aberrant DNA methylation are consistent with results obtained in vivo in mice subjected to a diet with extreme folic acid deficiency [29–31].

### Nuclear abnormalities and aneuploidy

The discovery of Howell-Jolly bodies in erythrocytes with megaloblastic anaemia was the first evidence of chromosome damage caused by folic acid deficiency [32–34]. Howell-Jolly bodies are chromosomal fragments that lag behind during anaphase, a form of micronuclei present solely in erythrocytes. Studies on sufferers of Chron disease found that their frequency was linked with low serum and intracellular folate [35].

Similar results have been obtained for human lymphocytes cultured in folic acid-depleted media. The formation of micronuclei and other nuclear abnormalities such as nucleoplasmic bridges and nuclear buds were observed, which are indicators of genomic damage [36, 37]. Under similar conditions, particularly in the absence of thymidine, fragile sites and chromosome breakage also occur, which are attributable to the misincorporation of uracil in the DNA [19, 20, 38–40]. Increased chromosomal damage and the inability to efficiently repair aberrant hypoxanthine bases was observed in deprived lymphocyte cultures when compared to folate-replete controls [22]. Accumulation of S phases and subsequent induction of apoptosis has also been described [41].

Wang et al. [42] and Beetstra et al. [43] revealed an association between folic acid deficiency and the incidence of aneuploidies of chromosomes 17 and 21, often observed in breast cancer and leukaemia. This was also observed for chromosome 8 [44], found abnormal in number in prostate, skin and breast cancers, cholesteatoma and leukaemia [45–48]. In particular, trisomy 8 has been reported as a recurrent chromosomal abnormality in acute myeloid leukaemia (AML) and hence considered a cytogenetic marker of AML [49, 50]. Ni et al. [44] demonstrated that aneuploidy of chromosome 8 is influenced by folic acid deficiency similarly to chromosome 17. In addition, riboflavin deficiency seemed not to aggravate the risk of aneuploidy, which is coherent with other studies in which riboflavin had no influence on the formation of micronuclei [51].

### Telomere abnormalities

The consumption of vital micronutrients through diet, most notably folic acid, represents an important determinant for the maintenance of telomere length and health [52–54]. Telomeres consist of repeated hexameric sequences (TTAGGG) found at the end of chromosomes together with other accessory proteins. This complex is called “telosome” and protects chromosome ends from degradation and chemical damage, which could result in chromosomal instability and breakage [55]. Because of their chemical composition rich in thymidine, chromosome ends are thought to be susceptible to uracil misincorporation and impaired repair.

Telomeric abnormalities and dysfunction have been associated with ageing, cancer and degenerative diseases [55–57]. Telomeric shortening is commonly observed in initial stages of tumorigenesis, but in some cancers, excessive telomerase activity can cause abnormally elongated telomeres [56]. Epigenetic changes, especially DNA methylation, can also disrupt the normal maintenance of the telomere length by interfering with the expression of the machinery involved [58–60]. These effects are particularly critical during foetal life, as early programming events in utero can have a permanent impact on health and susceptibility to disease.

Low maternal folic acid levels have been shown to cause shorter telomeres in the newborn, although the clinical implications in later life are not known [61, 62]. In population studies, low folate status was associated with telomere abnormalities in a non-linear manner. Considering that the normal concentration in population ranges between 13.5 and 45.3 nmol/L [63], with a plasma concentration below 11.6 nmol/L, telomere length increased, whereas shortening was observed above the median [64]. In vitro, human lymphoblasts cultured in folic acid-deficient medium showed that chromosomes undergo an initial telomeric elongation followed by a rapid shortening, both being indicators of genomic instability [54].

### Damage to mitochondrial DNA

Genomic instability is not limited to nuclear DNA damage, as also mitochondrial DNA (mtDNA) appears to be affected by lack of folic acid. Folates possess anti-oxidant properties against reactive oxygen species (ROS) and lipid peroxidation and are able to process harmful metabolites, preventing mitochondrial toxicity [9, 65, 66]. Folic acid deficiency can cause oxidative stress and initiate apoptosis [67] with deleterious consequences on the integrity of mtDNA. Most studies, conducted on rodents, found an increased number of mtDNA deletions when folic acid was deficient [68–72]. Studies on different tissue types in rats demonstrated that mtDNA deletions in the liver induced by ageing are associated to folate levels, indicating that folic acid supplementation reduces the occurrence of these deletions by two- to threefolds when compared to depleted rats [72]. This has also been observed in rat lymphocytes, where folic acid deficiency causes increased deletions by nearly fourfolds [71].

Accumulation of somatic deletions and mutations in the mtDNA may play a role in tumorigenesis, as mtDNA is subjected to detrimental factors originating from the environment, including dietary deficits [73]. Damage to mtDNA is capable of inducing reactions that can damage the nuclear DNA as well, by both genetic and epigenetic mechanisms, including methylation, chromatin remodelling and signalling pathways [74]. This is particularly relevant in cancer because damaged mitochondria can induce changes in the genome and in the surrounding microenvironment, both capable of creating an advantageous setting for tumorigenesis [75].

In haematological malignancies, somatic mutations and changes in mitochondrial gene expression have mainly been observed in myelodysplastic syndromes [76, 77]. Acquired mitochondrial mutations have also been found in the bone marrow of nearly 40% of patients with adult leukaemia, when compared to normal tissue with no mutations [78]. These results are similar to other studies on different cancers, where only a fraction of patients with the same malignancy showed mtDNA mutations [79–83].

Acquired mtDNA deletions and low folate status have been associated with incidence of hepatocellular carcinoma, suggesting that carcinogenesis is attributable to the deleterious effects of folate deficiency on the stability of both nuclear and mitochondrial DNA [84]. Studies on the impact of nutrients and correlated mitochondrial damage in the offspring are limited, but it is known that mtDNA in the placenta responds to environmental factors. For instance, airborne pollution is capable of damaging the mitochondria and altering methylation patterns, potentially leading to adverse health outcomes for both mother and foetus [85, 86].

The mitochondrial genome is highly variable among different populations, which poses a limitation when searching possible pathogenic mutations [87, 88]. Also, the consequences depend on the locus and the extent of the mutations, as certain mutations seem to have no effect on the function, metabolism or phenotype of the mitochondrion or the cell as a whole [78]. In fact, potentially pathogenic mutations are found in the population, although the vast majority is not clinically expressing the disease [89].

### Genomic damage caused by folate deficiency and ionising radiations

To better comprehend the extent of the DNA damage inflicted by folic acid deficiency, comparative studies have been conducted on the similarities with ionising radiation damage. Courtemanche et al. [90] showed that cultured lymphocytes depleted of folic acid or irradiated with high-dose radiation presented similar DNA double-strand breaks and were both subjected to decreased proliferation, cell cycle arrest and apoptosis. However, differences in gene expression analysis indicated that, although similar, the damage seems to arise following different pathways. The exact mechanism that the cell employs in response to nutritional deficits has been studied in *Caenorhabditis elegans* [91]. Folate deficiency also seems to be an enhancing factor of DNA damage resulting from radiation in vivo [92], which led to the investigation of folic acid acting as a radioprotective agent in vitro [93].

## Folates as modulators of DNA methylation

**Fig. 4:**
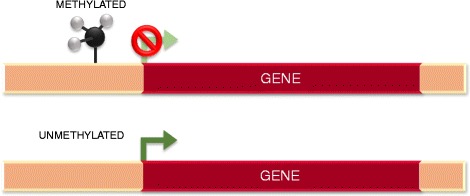
Methylation can modify the expression of genes. Methylated regions upstream the start site of transcription are a signal of gene repression. Conversely, the absence of methylation promotes the transcription of the gene

Methylation of CpG islands at the promoter or regulatory regions of a given gene represses its expression, whereas unmethylation allows the transcription to proceed (Fig. 4). The exact mechanism is not completely understood. The positioning of the methyl group is thought to physically impede the binding of the transcription machinery and block the activation of that gene. However, exceptions to this mechanism exist. For instance, methylation seems to activate the transcription of the gene for telomerase [94]. Hypo- and hypermethylation of CpG islands is of particular interest in carcinogenesis, as tumour-suppressor genes and proto-oncogenes become erroneously inactivated and activated, respectively, causing uncontrolled growth [95, 96].

DNA methylation forms distinctive patterns in different tissue types that can be transmitted to the next generation of cells to perpetuate the expression profile for the appropriate differentiation of tissues. However, it appears that the maintenance of the methylation per se is not sufficient to guarantee the stable expression pattern throughout the entire genome [97]. The poor availability of folates as a source of methyl groups can influence the ability to maintain the correct methylation patterns, causing mainly hypomethylation of genomic DNA [98, 99], reversible upon re-supplementation [17, 100–102]. The disruption of methylation patterns by lack of folates can also occur by hypermethylation of certain loci, although this might seem counterintuitive. Different cell types and specific loci in the genome respond to folate status in different ways, thus affecting gene expression by various mechanisms [16, 103, 104].

### Folates and DNA methylation patterns in foetal life

Folate deficiency has been shown to influence the methylation status of certain genes at birth in both animals and humans. Recent findings in rats clarified that gestational folic acid intake can influence the progeny’s gene expression and this occurs in an organ-specific manner, with the brain being the most susceptible to these changes [105].

This is particularly relevant in conception, which occurs before the DNA methylation pattern re-programming. The critical window period to determine the DNA methylation pattern is right after fertilisation, when the zygote is in between the morula and blastocyst phases [106]. The periconceptional period is also crucial for the establishment of correct genetic, epigenetic and metabolic settings for successful reproduction, and nutrients of the one-carbon metabolism play a pivotal role in these processes [107].

Gonseth et al. [108] demonstrated that exposure to folates 12 months before conception is capable of modifying DNA methylation in healthy newborns. Genes involved in neural crest development (*TFAP2A*), acute myeloid leukaemia (*STX11*), cystic kidney disease (*CYS1*) and other genes involved in foetal facial (*OTX2*) and neural development were sensitive to the modifying effects of folate deficiency on methylation patterns. Deficiency in periconceptional folate consumption was associated with methylation of promoter regions of these genes resulting in downregulation, with potential tumorigenic effects on genes with tumour-suppressor properties. Steegers-Theunissen et al. [109] examined the use of folic acid during pregnancy and the methylation status of the offspring. Maternal folic acid supplementation resulted in a 4.5% increase in the methylation of *insulin-like growth factor 2* (*IGF-2*) in the progeny, which is positively associated with maternal SAM levels, inversely proportional to weight at birth, and it has been linked with several chronic disturbances. Chang et al. [110] compared DNA methylation levels in different tissues from aborted foetuses affected by NTDs with normal healthy controls. The brain tissue was hypomethylated in foetuses with NTDs. The folate in serum was also lower in mothers whose foetuses presented NTDs, further confirming the association between folic acid levels and methylation status.

However, the hypothesis that folate deficiency acts as limiting factors during the embryonic development is controversial. In fact, studies on the association between the exposure to folates during foetal life and the global DNA methylation status at birth in folate-replete populations have yielded contrasting results. It is plausible that the relationship between folic acid consumption and its effects on DNA methylation is dose dependent, meaning that populations with severe folic acid deficiency are more prone to the modifying effects of folate on methylation than folate-replete populations [108, 111]. This is coherent with studies from Heijmans et al. [112] and Tobi et al. [113] who investigated the epigenetic alterations in individuals who suffered hunger during war with a severe deficiency in folic acid. Heijmans et al. found that *IGF-2* was hypomethylated, and Tobi et al. identified hypomethylation in *INSIGF* and hypermethylation in *IL-10*, *LEP*, *ABCA1*, *GNASAS* and *MEG3*, which are associated with growth and metabolic disorders.

### Contribution of diet in the epigenetic control of gene expression

Maternal diet during pregnancy can influence many physiological and metabolic functions in the foetus and determine susceptibility in the development of diseases later in life [114]. The intrauterine process of foetal programming has been associated with physical, psychological, metabolic and pharmacological stress factors. These exogenous events are capable of inducing permanent changes in the foetus, with potential post-natal consequences [115]. The developing foetus is subjected to an extensive programme of cell division, growth and differentiation. Differentiation requires a precise organisation of gene expression, which is regulated by DNA methylation, chromatin structure and other genetic and epigenetic determinants [116]. Any interference with these events of epigenetic modification can permanently compromise gene expression and have serious repercussions on the development of the organism [115, 117].

Experiments on agouti mice proved that nutrition can induce epigenetic changes in the offspring by interfering with normal DNA methylation, influencing the susceptibility to disease [118–120]. The coat colour of agouti mice is determined by the methylation of the *agouti* gene, which is strongly dependent on the maternal diet [120]. This has led to the hypothesis that the risk of developing diseases might partially be attributable to parental nutrition [121–123]. An intracisternal A particle (IAP) is present in the upstream region of the agouti gene. The gene is regulated by the promoter activity of IAP by methylation. The availability of methyl groups from the maternal diet during pregnancy increases the methylation of DNA in the IAP gene, inducing phenotypic changes that affect the gene expression of the progeny (i.e. change in coat colour) [118, 119]. In a separate study, Waterland et al. [121] identified similar loci in the human genome with distinct methylation patterns depending on the season of birth.

In mice, a paternal diet low in protein induced epigenetic changes in the progeny, when compared to controls with an equilibrated protein intake. The changes affected the methylation of DNA in the liver in the genes involved in the biosynthesis of lipids and cholesterol [124]. Paternal fat-rich diets reduce the methylation status of the *Il13ra2* gene in pancreatic cells in female progeny [59]. A protein-depleted diet in the mother during pregnancy has also been shown to dysregulate DNA methylation and gene expression but was reversible if folic acid was supplemented [114].

## Folates and cancer

The capability of folates of modulating DNA methylation, repair and synthesis suggests a role in tumorigenesis. Folate deficiency has been proposed as a contributing factor in the development of cervical, lung, breast, brain, colorectal and pancreatic cancers [6, 14, 125–127]. In particular, a large number of studies on humans have shown that a higher intake of folates through diet and higher plasma levels of folate are associated with lower risk of developing polyps and tumour in the colon [21, 128–131].

### Interaction of folates with cancer-related genes

It is now well established that mutations in the *BRCA1* and *BRCA2* genes can result in defective DNA repair and lead to the development of breast cancer. As folate deprivation is linked with chromosomal abnormalities, it has been proposed that carriers of germinal mutations to these genes are more susceptible to the genomic damage caused by low levels of folic acid, when compared to individuals without the mutation. The study revealed that the mutation of *BRCA1* and *BRCA2* did not increase the magnitude of damage caused by folic acid deficiency. Moderate folic acid deficiency showed a greater chromosomal instability than the damage observed in mutation carriers [132].

Lack of folic acid can also affect the genetic and epigenetic integrity of p53, which is a well-characterised tumour suppressor [29, 133]. Inactivating mutations of *p53* have been described in cancer [134]. However, it appears that structural damage to the gene caused by folate deficiency has no effect on gene transcription, expression and function and has not prevented folic acid-deprived lymphocytes from undergoing apoptosis via p53 activation [41, 135]. In rats, *p53* mRNA transcript levels in the colon were decreased by folic acid deprivation, but no changes were observed in its methylation status, suggesting that colon cancer tumorigenesis is initiated by other mechanisms than p53 inactivation [136].

### Excessive folic acid intake and cancer progression

It is generally agreed that high levels of folic acid confer protective action lowering the risk of malignant disease [137, 138]. According to the World Health Organization (WHO) guidelines regarding folic acid intake for pregnant women, over-supplementation has no negative outcomes on health [139], which has however been contested in a number of studies. Selhub and Rosenberg [140] discussed various issues in discordance with the claims from the WHO, while Smith et al. [98] highlighted the possibility of folic acid supplementation not being beneficial for the population as a whole. For instance, an excessive intake of folic acid may nourish tumours that have already initiated. The stage of the malignancy and the time period of folic acid supplementation could make a substantial difference in whether folic acid acts as suppressor of malignant transformation or promoter of growth for established tumours [21, 141]. This has been particularly evident for colorectal cancer [21, 142] and prostate cancer [143]. In vitro studies on colon cancer cell lines showed that folic acid supplementation in medium is capable of changing DNA methylation patterns, as well as altering the proliferative capacity and phenotype of these cells in culture. This is of particular importance in understanding the role of folic acid supplementation on the behaviour of tumours in terms of phenotypic changes, motility and invasion [144]. Other studies have reported an increased proliferation due to altered DNA methylation in response to excessive folic acid supplementation [6, 145].

## Folate intake, haematopoiesis and leukaemia

### Folate in haematopoiesis

Folic acid and vitamin B12 are involved in the correct production and maturation of blood cells from haematopoietic stem cells (HSCs), particularly for the production of red blood cells [146]. Bills et al. [147] proved that folic acid-depleted mice showed an ineffective haematopoiesis, with changes affecting the maturation of progenitor cells.

This is evident in megaloblastic anaemia, a disorder of erythropoiesis that arises from folic acid or vitamin B12 deficiency. This haematological disorder is characterised by the accumulations of enlarged, immature erythroblasts in the bone marrow due to an impaired DNA synthesis and capacity to divide [148], highlighting the role of these vitamins in the maintenance of genomic health.

### The prenatal origin of leukaemia

**Fig. 5:**
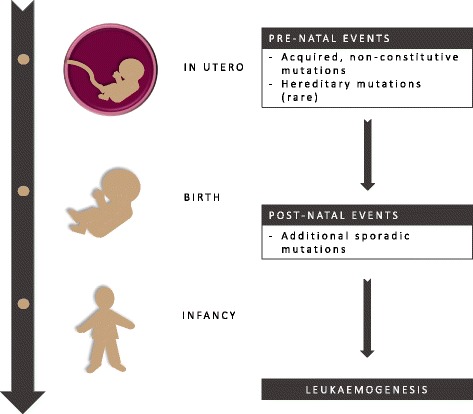
Multi-hit hypothesis for the insurgence of childhood leukaemia. The initiation of childhood leukaemia requires multiple oncogenic events to occur, with the first event occurring in utero and a second hit occurring after birth or later in life. The first event may be called the predisposing condition at the genomic level that is necessary but not sufficient for the insurgence of cancer. Leukaemia is initiated when a second event promotes abnormal cell proliferation

Leukaemia is characterised by the presence of acquired chromosomal rearrangements that are confined to the diseased bone marrow cells. Retrospective studies carried out on Guthrie cards of individuals that developed leukaemia during childhood showed the presence of genetic rearrangements in those archival samples. This constitutes the proof that the initiating genetic events leading to leukaemia were already present in utero [149, 150]. Furthermore, secondary events must occur after birth to promote the cancer through clonal expansion [151]. Notwithstanding, the exact aetiology of childhood leukaemia is largely unknown. The causes for the arising of chromosomal translocations and the methylation patterns that accompany different leukaemia phenotypes are still not fully understood. Leukaemogenesis appears to be a result of genetic and environmental factors, occurring prior and during pregnancy, but also post-partum and later in life (Fig. 5). Several environmental factors have been identified, including exposure to radiation, certain chemicals or infections [152, 153]. The role of nutrition is also gaining importance, as micronutrients are capable of interfering with the genomic stability, and studies are already being undertaken to assess the extent of this influence [154, 155].

### Maternal intake of folic acid and childhood leukaemia

**Fig. 6:**
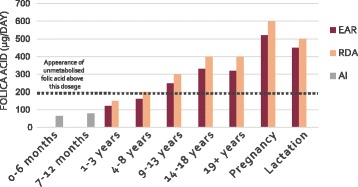
Current recommendations for folic acid nutritional requirements per age. The Institute of Medicine (Food and Nutrition Board) determined the recommended daily assumption (*RDA*) and Estimated Average Requirements (*EARs*) for folate according to age and status. For infants (0–12 months), adequate intake (*AI*) is presented instead, as the influence of maternal nutrients can interfere with experimental evidence for RDA and EAR. During pregnancy and lactation, the requirements are higher, as the developing foetus needs high levels of folic acid. At doses above 200 μg, unmetabolised folate is detectable in plasma

The current recommended consumption and supplementation of folic acid intake is summarised in Fig. 6. The recommended daily assumption (RDA) of folic acid is 400 μg/day in adults, 600 μg/day in pregnant women and 500 μg/day during lactation. The Estimated Average Requirements (EARs) are 320, 520 and 450 μg/day, respectively [5]. Folic acid supplementation is required during pregnancy, first and foremost for the prevention of NTDs [156]. It appears that consumption of folic acid and multivitamin supplements reduces the risk of leukaemia in the offspring, one of the most prevalent cancers in children under 15 years of age [157]. Although research on this topic has been contradictory [158–162], recent publications suggest that folic acid does play a protective role against these malignancies.

Metayer et al. [163] considered 12 studies conducted in ten countries from 1980 to 2012 to extract data on the intake of folic acid and other vitamins in women during and before pregnancy. According to this epidemiological study, the risk of acute lymphoblastic leukaemia (ALL) is lowered when folic acid plus multivitamins were taken during the year before and during pregnancy. Similarly, the risk for AML decreased with folic acid intake before and during pregnancy, but other vitamin intake seemed not to have the same protective effect. In particular, folic acid supplementation seemed to have a more evident protective effect towards AML than ALL, with a diminished incidence of 32% for AML versus 21% for ALL. Vitamin intake was associated with a decreased risk for ALL of 15%, whereas no correlation was found with the risk of developing AML. Despite the lack of information on dosage, it is clear that folic acid and other vitamins are required during the entire course of pregnancy, from the very early stages, as has long been known in regard to NTDs [156]. At doses above 200 μg/day, unmetabolised folic acid is observed in circulation [164]. Since the long-term effects of this phenomenon have not been investigated, the recommended dosage (Fig. 6) remains questionable, as well as the potential risks of the metabolite in plasma [140, 165, 166].

In a recent matched case-control study from Singer et al. [167], maternal intake of folate, vitamins B12 and B6, riboflavin and methionine 1 year before pregnancy were examined, confirming their protective action against ALL and AML. Similar findings are shown in a more comprehensive study focusing on the maternal dietary quality as a whole, rather than individual nutrients [168]. Other case-control studies [169] have investigated the risk for ALL only, the most common form of childhood leukaemia, finding reduced risk for the disease linked to maternal supplementation of folic acid.

### Role of genetic polymorphisms in the development of childhood leukaemia

Polymorphisms in genes participating in folate metabolism are capable of modifying the pathways through which folic acid is processed but also influence the susceptibility to cancer (leukaemia, lung, breast, brain, colorectal, gastric, head and neck malignancies) and other diseases. Mutations have been identified in the *MTHFR*, *TS*, *MTR* and *MTRR* genes (Fig. 2) [170]. The most common variants of the *MTHFR* gene are C677T, characterised by a C → T transition, and A1298C, where an A → C transversion occurs; both result in reduced enzymatic activity [171, 172].

A number of studies have been undertaken to understand the role of polymorphisms in genes involved in folic acid metabolism in the development of childhood leukaemia, yielding discordant results. While a reduced susceptibility to ALL has been disproven by some [173–178], others showed a significant decrease in its insurgence linked to the presence of polymorphisms [179–182]. Five studies on the *MTHFR* 677CT polymorphism did not find any significant difference in the susceptibility to ALL [173–177], whereas four studies proved that the polymorphism conferred a diminished risk in the development of the malignancy in the Brazilian and Western European populations [179–182]. Similar results come from the *MTHFR* 677TT variant. While most studies agree on an insignificant difference in risk for ALL, the Korean population showed to have an increased susceptibility due to the polymorphism [173, 176, 182].

The A1298C variant shows similar conflicting results. Some data suggest that it plays a role in increasing the risk for childhood leukaemia [182, 183], although this has been disproven by others [173, 176, 180]. The *MTRR* A66G polymorphism seemed to confer a reduced risk of developing ALL in most populations apart from the Korean population, where the variant did not show to influence the susceptibility to the disease [184]. Polymorphisms of the *TSER*, the promoter enhancer region of the *TS* gene, did not show to be a significant factor in determining the risk for ALL [185–188].

Milne et al. [161] investigated the risk of ALL in a population-based case-control study, in which 392 individuals with polymorphisms in the *MTHFR*, *MTR*, *MTRR* and *CBS* genes and 535 controls were analysed, investigating both parents and progeny. The risk of developing ALL seemed to be diminished in offspring of fathers with the genotype *MTRR* 66GG. The authors concluded that the risk of ALL in the progeny due to polymorphisms in genes of the folate metabolism can be influenced by maternal intake of folic acid, although more research is needed.

Limited information is available about the susceptibility to AML. Most studies conclude that there is no association between polymorphisms of *MTHFR* and the risk of AML [189–191]. Certain studies, however, have proven that the polymorphic variant *MTHFR* C677T is a risk factor for the development of AML in the Romanian and Asian populations [192–195].

The differences in these results could be attributable to genetic and environmental variants in geographic areas and populations [178, 196].

Most studies on the risk of leukaemia in children have focused on the role of single nutrients, rather than a wider understanding of the maternal diet as a whole. It is likely that several factors and interactions of nutrients are accountable for the development of the disease. Further research is needed to uncover the multiple aspects of the diet and their effects on the health of the mother and progeny. Paternal periconceptional folic acid supplementation has also been considered in some studies, but results are inconclusive [197, 198].

## Conclusions

Correct nutrition represents one of the most crucial protective factors for many pathological conditions including cancer. Certain nutrients have proven to detain a protective and preventive role for the maintenance of human health. However, the interaction between specific nutrients and development of disease is complex and might be influenced by additional dietary and environmental factors, indicating that the association between maternal folic acid intake and methylation status of the progeny is non-linear and non-definitive.

Folates are required within the cell for synthesis, repair and methylation of nuclear and mitochondrial DNA. The studies examined in this review suggest that folic acid deficiency is capable of interfering with these processes, which are extremely important during foetal development and, if altered, are capable of promoting carcinogenesis and the development of other diseases. In particular, DNA damage due to lack of folates can lead to the formation of chromosomal abnormalities, which are considered a hallmark in cancer and leukaemia. Overall, folic acid intake during pregnancy seems to provide protection against the risk of leukaemia in the offspring.

The exact optimal dosage is still unclear, considering that excessive intake of folic acid might have serious drawbacks, including the nourishment of pre-existing cancers or pre-cancerous conditions. However, the overall dietary plan in pregnancy, disease and non-disease conditions should consider the interaction of multiple nutrients, rather than the accurate dosage of single compounds. Genomic damage and cancer growth can be potentially controlled through a combination of different micronutrients and correct dosage. In this respect, personalised nutrition could be implemented not only to provide a better diet plan during pregnancy but also as an adjuvant to anti-cancer therapies for specific tumours.

## Abbreviations

AI: Adequate intake
ALL: Acute lymphoblastic leukaemia
AML: Acute myeloid leukaemia
dTMP: Deoxythymidine monophosphate
dUMP: Deoxyuridine monophosphate
EARs: Estimated Average Requirements
HSCs: Haematopoietic stem cells
IAP: Intracisternal insertion A particle
mtDNA: Mitochondrial DNA
NTDs: Neural tube defects
RDA: Recommended daily assumption
ROS: Reactive oxygen species
SAM: S-Adenosylmethionine
THF: Tetrahydrofolate
WHO: World Health Organization
